# Optimizing Habitat Management to Boost Beneficial Insect Populations for Effective Rice Pest Control

**DOI:** 10.3390/insects17080754

**Published:** 2026-07-23

**Authors:** Gebretsadik Kifle Gebreegziabiher, Shuyan Kou, Xianghong Shen, Xiang Li, Xu Yang, Songqiang Li, Binbin Huang, Cheng Jiang, Weihua Liu, Zhenhua Zhu, Pingrong Yuan, Zhigang Wu, Ping Huang

**Affiliations:** 1 Institute of Food Crops, Yunnan Academy of Agricultural Sciences, Yunnan Key Laboratory for Rice Genetic Improvement, Kunming 650201, China; kiflegeb2020@gmail.com (G.K.G.); sy_kou@yaas.org.cn (S.K.); 2021204023@stu.njau.edu.cn (C.J.); lwhynu@yaas.org.cn (W.L.); zhzhh86@126.com (Z.Z.); yuanpr2003@aliyun.com (P.Y.); 2 Tigray Agricultural Research Institute, Mekelle 492, Tigray, Ethiopia; 3 Yuxi Academy of Agricultural Sciences, Yuxi 653100, China; 13887723260@163.com (X.S.); lx19840215@163.com (X.L.); 4 Yunnan Provincial General Station of Agricultural Technology Extension, Kunming 650201, China; ynsnjtgz@163.com; 5 Yuanjiang Hani, Yi and Dai Autonomous County Agricultural Development Service Center, Yuxi 653100, China; lsq584131@126.com (S.L.); 18387542539@163.com (B.H.)

**Keywords:** rice, natural enemies, habitat management, ecological engineering, integrated pest management, conservation biological control, rice pests

## Abstract

Rice is a staple food for more than half of the global population, but its production is threatened by insect pests such as planthoppers, stem borers, leafhoppers, and defoliators. The widespread use of chemical pesticides has raised concerns regarding environmental contamination, pest resistance, and negative effects on beneficial arthropods. This review examines the role of biological control and habitat management in sustainable rice pest management. It highlights the diversity and ecological roles of natural enemies, including parasitoid wasps, mirid bugs, ladybird beetles, and spiders, which help suppress pest populations in rice ecosystems. Key habitat management approaches, such as conservation biological control, ecological engineering, banker plant systems, flower strips, and the preservation of non-crop habitats, are discussed for their capacity to enhance the abundance and effectiveness of natural enemies. The review also considers integrating these approaches into IPM programs to reduce reliance on pesticides while maintaining crop productivity. Evidence from rice-growing regions demonstrates the effectiveness of habitat-based strategies in improving pest suppression and strengthening ecosystem resilience. However, challenges related to landscape complexity, farmer adoption, long-term implementation, and the need for a clear roadmap for future landscape-scale entomological research remain. Future research should focus on optimizing habitat management practices and integrating ecological approaches with modern agricultural technologies to support sustainable rice production.

## Simple Summary

Rice production is severely affected by insect pests such as planthoppers, stem borers, leafhoppers, and gall midges, which cause major yield losses worldwide. Beneficial insects and other natural enemies, including spiders, ladybird beetles, dragonflies, damselflies, and parasitoid wasps, play a key role in suppressing these pests and reducing reliance on chemical insecticides. This review highlights how habitat management and ecological engineering can strengthen biological control in rice ecosystems. Practices such as maintaining non-crop vegetation, flower strips, banker plants, cover crops, and shelter habitats provide food, alternative hosts, and refuges that enhance the abundance and effectiveness of natural enemies. Increasing biodiversity and landscape heterogeneity generally improves pest suppression, lowers pesticide use, and supports sustainable rice production. Several case studies demonstrate that ecological engineering and conservation biological control can successfully reduce populations of major rice pests, particularly brown planthoppers and stem borers. Overall, the review concludes that habitat management and ecological engineering are essential components of IPM. By conserving and enhancing beneficial insects, these approaches offer environmentally friendly, sustainable solutions to improve rice productivity while reducing reliance on synthetic pesticides. Finally, we outline technical recommendations and future research trajectories to facilitate the integration of habitat manipulation into standard rice integrated pest management frameworks globally.

## 1. Introduction

Rice (*Oryza sativa* L.) is one of the world’s three major staple food crops, alongside maize and wheat, and serves as the primary food source for more than half of the global population [1,2]. Approximately 90% of global rice production occurs in Asia, with China and India being the leading producers and consumers of this essential staple crop [3]. China is currently the world’s largest rice producer [2]. Rice is typically grown annually using transplanted seedlings or direct seeding in flooded or irrigated fields [4,5]. Bunds separate fields, retain water, and provide habitat for beneficial organisms [6]. The rice plant progresses through stages of seedling establishment, vegetative growth, reproductive development, grain filling, and maturation before harvest [6,7]. After harvest, crop residues are either removed or returned to the fields. Despite its global importance, more than 20% of rice production is lost annually to insect pests [8,9], resulting in significant yield losses and reduced crop quality. The major pest groups include planthoppers [10], generalist leafhoppers, true bugs, and locusts [9], as well as the African rice gall midge, armyworm, and rice stem borers [11]. These pests are controlled by natural enemies, including generalist predators such as spiders and predatory bugs, which can disperse widely, and specialist parasitoids that usually spread over shorter distances [12].

To advance sustainable agroecology, this review explicitly addresses core entomological questions regarding how specific predatory and parasitic insect taxa respond behaviorally, numerically, and functionally to intentional vegetation modifications. Specifically, we evaluate: (1) How do specialized planthopper egg parasitoids navigate localized landscape heterogeneity to achieve optimal parasitism rates? (2) What nutritional and volatile cues govern the recruitment of beneficial predatory insects to diversified flower strips? (3) What mechanisms dictate the microclimatic suitability of winter shelters for stabilizing beneficial arthropod populations across cropping seasons? By shifting the analytical focus from generalized biocontrol concepts to specific insect biology and trophic dynamics, this review aims to provide rice entomologists with actionable, eco-physiologically grounded blueprints for conservation biological control. This aligns with recent bibliometric assessments and regional integrated pest management frameworks emphasizing structured, landscape-scale entomological designs [11,13]. 

Beneficial insects and other arthropods play crucial roles in providing ecosystem services that help manage rice pest populations and reduce dependency on chemical pesticides [14,15,16]. Key predators, such as ladybird beetles, spiders, dragonflies, damselflies, rove beetles, and mirid bugs, as well as parasitoids such as *Anagrus* spp. and *Trichogramma* spp., are fundamental to regulating herbivore populations in rice ecosystems [16,17]. These natural enemies mitigate pest populations through predation and parasitism, thereby supporting integrated pest management strategies and decreasing reliance on synthetic pesticides [18,19,20]. However, the success of biological control largely hinges on the availability of appropriate habitats, food resources, and refuges to sustain natural enemy populations throughout the cropping season [6,21]. 

Currently, rice agroecosystems are undergoing significant changes characterized by reduced crop diversity and the loss of semi-natural habitats [8,12,22]. Habitat management and ecological engineering have emerged as promising approaches for enhancing biological control in rice-based agroecosystems [23,24]. These strategies focus on increasing biodiversity through practices such as intercropping, conservation of non-crop vegetation, establishment of flower strips, use of banker plants, and preservation of semi-natural habitats [12,25]. Banker plants are non-crop plants intentionally established within or adjacent to rice fields to provide alternative hosts, prey, or floral resources that maintain predator and parasitoid populations during periods of low pest abundance, thereby enhancing long-term conservation biological control [26,27]. By offering nectar, pollen, alternative prey, shelter, and overwintering sites, these practices strengthen natural enemy communities and enhance their efficacy in pest suppression [6,28]. Numerous studies have shown that diversified agricultural landscapes can increase the abundance and effectiveness of predators and parasitoids, resulting in reduced pest pressure, lower insecticide use, and improved crop yields [8,13,23,24].

The success of predatory arthropods is affected by factors such as habitat complexity, availability of alternative prey, and disturbances caused by broad-spectrum insecticides [16,29]. Among these natural enemies, spiders are particularly important because, as generalist predators, they capture large numbers of prey throughout their lifetimes and contribute substantially to biological pest control [16,30]. Higher spider diversity contributes to pest suppression as species vary in their hunting strategies and can target a broader range of pests [30,31]. However, because spiders are generalist predators, they may also consume beneficial arthropods, including predators and parasitoids, leading to intraguild predation that can reduce natural enemy populations and, under certain conditions, weaken biological control [6]. Nevertheless, field studies show that the overall impact of spiders in rice ecosystems is positive, as their pest suppression outweighs any negative effects on other natural enemies. Therefore, understanding the ecological and environmental factors influencing predator populations is essential for optimizing their roles in sustainable agriculture [6,32]. 

Within IPM, pest control decisions are guided by the economic injury level (EIL), the lowest pest density at which the cost of pest damage equals the cost of control, and the economic threshold (ET), the pest density at which management actions should be taken to prevent populations from reaching the EIL [33,34]. Conservation biological control aims to maintain pest populations below these thresholds rather than eradicate them. Consequently, low pest densities and minor feeding damage are often acceptable because they seldom reduce grain yield or economic returns [35,36]. In rice, cosmetic leaf injury generally has limited economic significance compared with damage occurring during critical growth stages or injury to reproductive structures that directly affects grain production [37,38]. Therefore, effective biological control can provide economically sustainable pest management even when minor visible pest damage remains.

We argue for the role of beneficial insects and habitat management in sustainable rice pest management. It underscores the diversity and ecological roles of key natural enemies, discusses habitat-manipulation strategies to improve biological control, and assesses ecological-engineering approaches within the context of IPM. Additionally, it presents case studies highlighting successful habitat-based pest management practices and identifies key challenges and future research directions to foster resilient and environmentally sustainable rice production systems.

## 2. Rice Pests and Beneficial Insects in Rice Ecosystems

### 2.1. Common Insect Pests of Rice

Rice production is severely constrained by a wide range of insect pests, including stem borers, hoppers, defoliators, gall midges, and several coleopteran pests [39]. Globally, rice planthoppers (RPHs) represent the most economically destructive pest complex, primarily consisting of the brown planthopper (BPH), *Nilaparvata lugens* (Stål); the white-backed planthopper (WBPH), *Sogatella furcifera* (Horváth); and the small brown planthopper, *Laodelphax striatellus* (Fallén) [10]. These hemipteran pests feed on plant vascular sap and can transmit viral diseases such as RYMV [40]. Lepidopteran stem borers, including *Scirpophaga incertulas* (Walker) and *Chilo suppressalis* (Walker), are similarly devastating; regional meta-analyses demonstrate that every 1% increase in "whiteheads" triggers a 4% decline in grain yield [40,41]. While conventional production intensification relies heavily on synthetic nitrogen inputs and broad-spectrum chemical sprays, these practices inadvertently improve pest nutritional quality while decimating the natural enemy communities that naturally suppress outbreaks [42]. To optimize the core arguments, an exhaustive taxonomic inventory of regional secondary pests has been omitted here in favor of focusing on these primary, globally representative threat groups.

### 2.2. Beneficial Insects in Rice Ecosystems

Natural enemies are key regulators of rice pests and can be broadly classified into generalist predators and specialist parasitoids [43]. Generalist predators, including spiders, dragonflies (e.g., *Crocothemis servilia* (Drury) and *Orthetrum sabina* (Drury)), damselflies (e.g., *Agriocnemis femina* (Brauer)), and ladybird beetles (e.g., *Micraspis discolor* (Fabricius) and *Harmonia octomaculata* (Fabricius)), suppress a wide range of pest species throughout the rice-growing season [44]. For instance, studies have shown that the experimental exclusion of generalist predators can lead to catastrophic pest outbreaks, with target pest populations increasing by up to 13-fold [43]. Conversely, specialist microhymenopteran egg parasitoids, such as *Anagrus nilaparvatae* Pang & Wang (Hymenoptera: Mymaridae) and *Pseudoligosita yasumatsui* Viggiani & Subba Rao (Trichogrammatidae), attack specific hosts and can achieve natural parasitism rates of up to 70% in undisturbed rice fields [45]. Detailed target pests and mechanisms for these primary taxa are systematically summarized in Table 1.

#### 2.2.1. Factors Threatening Natural Enemies

Beneficial arthropod communities face severe compounding threats from both anthropogenic practices and environmental stressors. Long-term field surveys indicate that dragonfly and damselfly populations are declining at an annual rate of approximately 14%, resulting in an overall 90% decimation over the past two decades due to the widespread adoption of systemic insecticides in aquatic rice systems [46]. Additionally, since rice is an annual monoculture that is harvested in the fall, beneficial insects experience a sudden decline in the availability of alternative prey. This resource scarcity is severely exacerbated by low winter temperatures, which drive high mortality among epigeal spiders and ladybird beetles if adequate vegetated overwintering microhabitats are absent [8]. 

#### 2.2.2. Synergistic Interactions Among Natural Enemies

Optimizing biological control requires understanding the predator–parasitoid synergistic relationships that occur within the rice canopy and benthos [47,48]. Generalist predators, such as mirid bugs and spiders, and specialized egg parasitoids, such as *Anagrus* spp., often attack different life stages of rice planthoppers simultaneously [47]. Although intraguild predation may occur, structurally complex habitats help reduce interference among natural enemies [47,48,49]. For example, web-building and ground-dwelling spiders primarily prey on planthopper adults and nymphs, lowering pest populations and reducing crop damage [31,50]. This enables egg parasitoids to parasitize the remaining host eggs more effectively. Together, these complementary foraging strategies enhance biological control and promote stable pest suppression throughout the rice-growing season [47,48].

**Table 1:** Representative natural enemies in rice agroecosystems and their functional roles.

Natural Enemy Taxon	Functional Group	Primary Target Pests	Mechanism of Action	Reference
*Crocothemis servilia* (Drury)	Generalist Predator	Planthoppers, leafhoppers, snails	Nymphs and adults prey on active immature and adult insect pests	[13]
*Harmonia octomaculata* (Fabricius)	Generalist Predator	Aphids, whiteflies, jassids, mites	Foliar search and consumption of soft-bodied insect cohorts	[43,51]
*Pardosa pseudoannulata* (Bösenberg & Strand)	Generalist Predator	Brown planthopper, leafhoppers	Active ground and lower-canopy hunting in field interiors	[52]
*Anagrus nilaparvatae* (Pang & Wang)	Specialist Parasitoid	Brown planthopper eggs	Endo-parasitoid oviposition into host eggs, causing host mortality	[1]
*Trichogramma* spp.	Specialist Parasitoid	Stem borers, leaf folders	Solitary or gregarious egg parasitism of lepidopteran targets	[1,39]

## 3. Principles of Habitat Management for Conservation Biological Control

CBC is a science-based approach that enhances pest management by conserving and promoting natural enemies within agroecosystems [21]. As a key component of IPM, CBC offers a more sustainable alternative to excessive chemical pesticide use and helps mitigate the negative effects of agricultural intensification on beneficial organisms [48,53,54]. This approach relies on habitat manipulation and modifications in agricultural practices to improve the growth, survival, and effectiveness of natural enemies [21,28]. Common mechanisms underlying enhanced pest suppression include the provision of alternative food resources such as nectar and pollen, alternative prey or hosts, and shelter [21]. 

Habitat management aimed at conserving natural enemies is fundamental to sustainable pest management and is based on ecological principles that promote stable biological control in agroecosystems [55]. Key strategies include intercropping, no-till farming, preserving non-crop vegetation, cultivating flower strips, increasing connectivity between croplands and unmanaged habitats, and using selective insecticides [21,56,57]. In addition, the introduction of plant-derived resources, including nectar, pollen, shelter, and alternative prey, creates favorable conditions that improve the fecundity, longevity, and persistence of natural enemies [6,48]. By providing access to these essential resources, habitat management enhances the fecundity, longevity, and persistence of natural enemies, thereby reducing reliance on chemical pesticides and promoting sustainable agricultural practices [55,58,59]. 

Numerous studies have shown that increased plant diversity enhances pest management and strengthens ecosystem services by restoring the natural components of agroecosystems [60,61]. Diversified agricultural landscapes generally support greater abundance and diversity of beneficial organisms, thereby improving the effectiveness of natural enemies [62]. Biodiversity contributes significantly to ecosystem services by restoring the natural components of agroecosystems [60,63]. Among the various diversification practices, intercropping—defined as the cultivation of two or more crops within the same field during a single growing season—has emerged as an effective non-chemical strategy for enhancing biodiversity and suppressing pests [60]. By supporting a broader range of natural enemies, intercropping reduces pest densities, lowers pesticide costs, and can increase crop yields and economic returns [64,65]. Furthermore, optimizing intercropping systems requires a better understanding of crop genotypic diversity, natural enemy diversity, and the role of VOCs in mediating plant–insect interactions [66,67].

### 3.1. Biodiversity Enhancement

Agricultural landscapes are increasingly being simplified through crop homogenization and the loss of semi-natural habitats, threatening arthropod natural enemies and disrupting ecosystem functions [8,68]. As agricultural practices evolve, changes in landscape structure can influence natural pest regulation, pesticide use, and biodiversity conservation, highlighting the need to enhance habitat diversity within farming systems [8,13]. 

Enhancing plant diversity in and around rice fields creates habitats and food for beneficial insects, like predators and parasitoids [8,25]. This can be achieved through practices such as intercropping, crop rotations, and planting cover crops [13,69]. Increased crop diversity supports generalist predators, including ladybird beetles, predatory bugs, and spiders, by ensuring a continuous supply of resources [8,70,71]. Numerous studies have shown that habitat diversification strengthens biological control in rice ecosystems. For example, increasing landscape diversity enhances parasitoid populations, while incorporating nectar-rich flowering plants promotes natural enemies and reduces crop damage [63,72,73]. Similarly, maintaining fallow lands and fragmented habitat patches can reduce insecticide dependence and contribute to sustainable pest management, particularly by supporting ladybird beetle populations [13]. 

However, biodiversity enhancement does not always lead to improved biological control [74,75]. Increased crop diversity may adversely affect parasitoids adapted to monocultures and, in some cases, favor pest outbreaks by concentrating host plants [9,76,77]. In addition, insect pests and their natural enemies frequently move between crop fields and adjacent semi-natural habitats, influencing their population dynamics [9,78]. Semi-natural habitats such as flower strips, forests, and grasslands provide resources for natural enemies but may also serve as refuges for pests [79,80,81]. Landscape characteristics such as smaller field sizes can facilitate the movement of natural enemies into crop fields [9,70,78]. Consequently, effective biological control requires a balanced combination of crop diversity and semi-natural habitats, with landscapes containing approximately 20–50% semi-natural areas generally supporting the highest abundance of natural enemies [8,70]. 

### 3.2. Non-Crop Habitat Management

**Figure 1:**
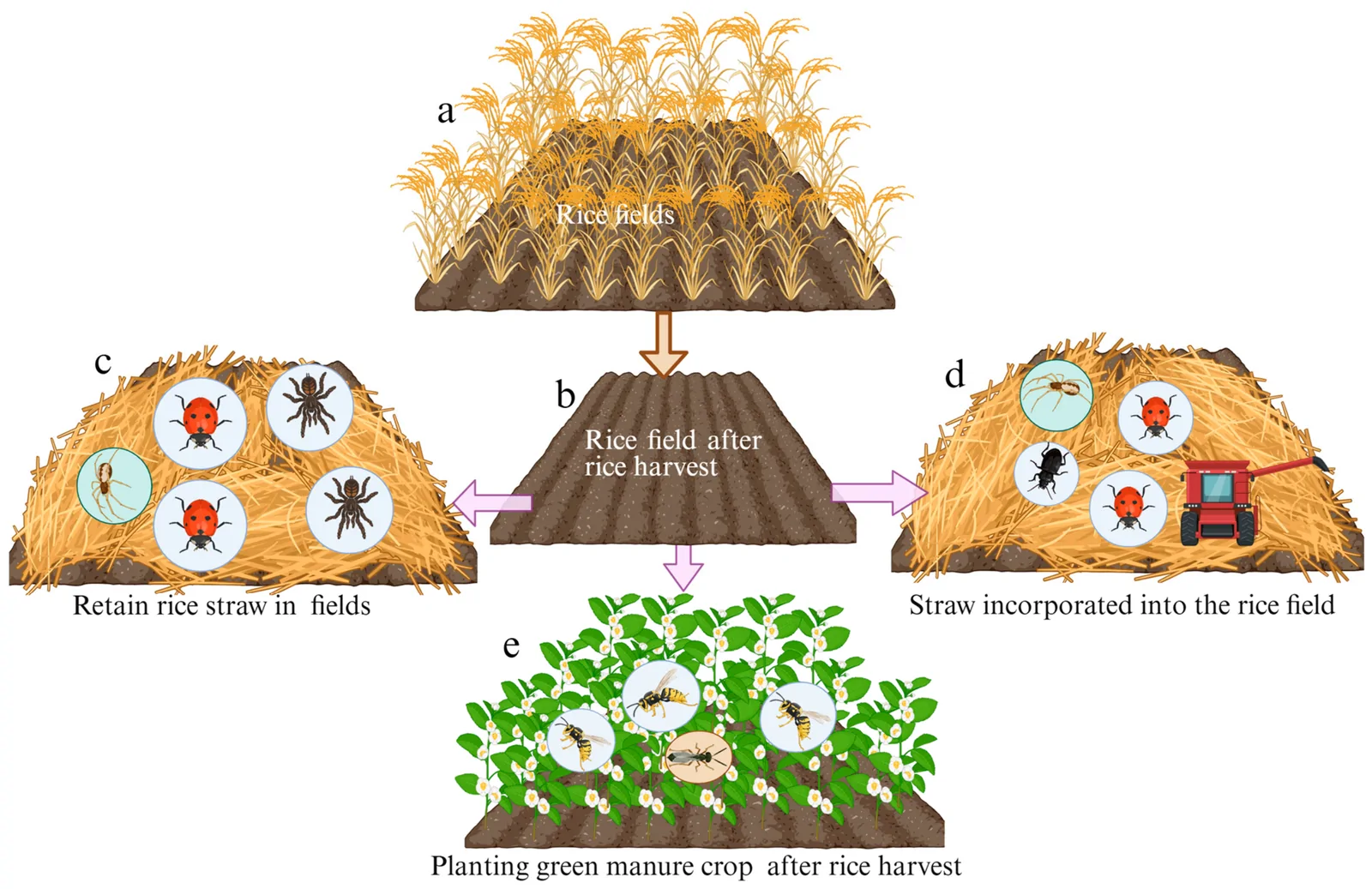
Practices that provide shelter for natural enemies in rice fields: (**a**) rice fields before harvest; (**b**) rice fields after harvest; (**c**) retained rice straw provides habitat and overwintering refuge for native predators; (**d**) incorporation of rice straw into rice fields enhances in-field refuges for natural enemies; (**e**) planting the green manure crop after rice harvest provides food resources and shelter for natural enemies during overwintering.

**Figure 2:**
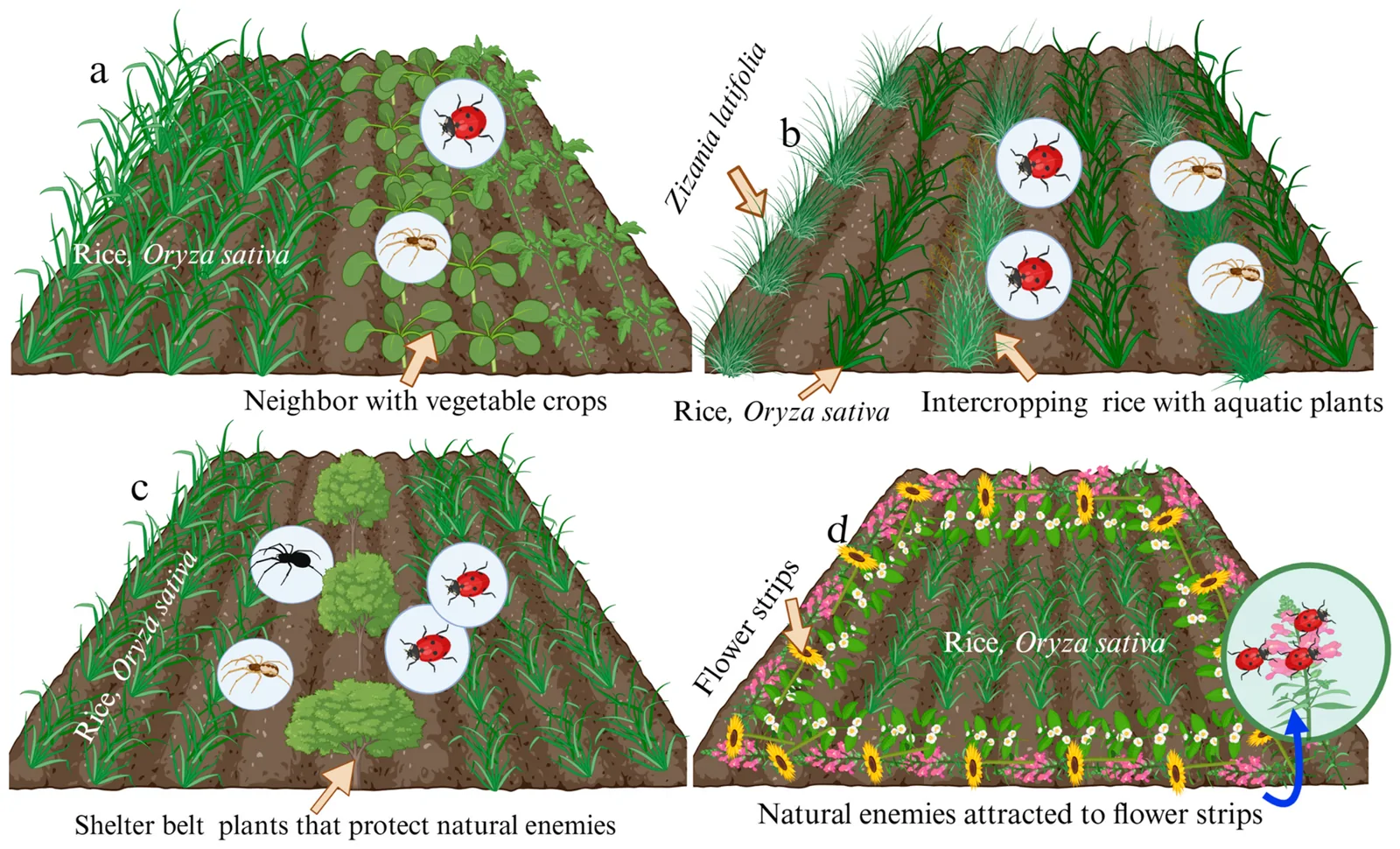
Types of shelters that support natural enemy conservation by providing food resources and alternative hosts: (**a**) planting diverse vegetable crops adjacent to rice fields enhances natural enemy abundance and diversity, improving pest suppression within and around the fields; (**b**) intercropping rice with aquatic plants such as *Zizania latifolia* increases habitat complexity and provides alternative resources for natural enemies; (**c**) natural shelter belts provide specific plant resources, diverse vegetation, and litter that protect natural enemies; (**d**) sowing flower strips along field bunds supply essential resources that improve the survival, re-production, and biocontrol effectiveness of predators and parasitoids.

Diverse non-crop habitats in agricultural areas provide homes for natural enemies, which play a crucial role in pest control within farming systems [82,83]. These habitats protect natural enemies from insecticides and provide food sources like nectar and pollen, as well as shelter and alternative hosts during winter [6,83] (Figure 1 and Figure 2). Research indicates that landscape complexity and diversity (i.e., compositional and configurational heterogeneity) significantly influence the abundance and effectiveness of these natural enemies [83,84,85]. However, farms with limited non-crop habitat often experience weaker pest control due to biodiversity loss, which disrupts food chains [83]. 

Distinguishing the effects of natural enemy communities in simple versus complex landscapes is challenging, as declines in abundance and diversity often occur simultaneously. However, research has shown that increasing biodiversity can enhance pest control [61,83,86]. For instance, greater compositional diversity in rice landscapes has been linked to increased abundance, richness, and biological control services provided by parasitoids, such as parasitic wasps [13,73]. Moreover, incorporating nearby nectar-rich flowering plants can boost the abundance of natural enemies and reduce crop damage [25,72] (Figure 2). In agricultural ecosystems that do not rely on chemical pesticides, naturally occurring populations of beneficial organisms significantly aid farmers by providing essential pest control services [13]. 

Evidence of the importance of natural enemies comes from experiments where excluding them with cages led to a significant increase in BPH populations, resulting in a 15.3–37.0% higher grain yield loss [13]. In contrast, uncaged plots and experimental fields maintained low BPH levels, thanks to the presence of natural predators, such as ladybird beetles and spiders, which were frequently observed attempting to enter the cages [13]. Maintaining natural vegetation around rice fields, such as field margins and hedgerows (typically within 0–5 m of the field edge) [6], can enhance biodiversity and create refuges for beneficial insects [87,88]. These areas serve as breeding grounds and provide shelter and food resources.

### 3.3. Ecological Engineering for Pest Management 

Ecological engineering is an eco-friendly, effective approach that involves designing and managing agricultural landscapes to enhance natural enemies and suppress pests within an agroecosystem [6,11]. This approach is grounded in ecological principles that strengthen biological control services while reducing reliance on external inputs, such as insecticides, thereby safeguarding biodiversity [89]. Important practices include establishing cover crops, conserving hedgerows, and maintaining buffer areas that offer shelter and supplementary food resources for beneficial insects [11,90]. In addition, landscape diversification strategies, including crop rotation and intercropping, help break pest life cycles and lessen their adverse effects on rice production [11,48,91]. Non-crop species are added to supply essential resources—nectar, pollen, and alternative prey—and create stable habitats that fill temporal and spatial resource gaps in simplified landscapes [18,92] (Figure 2). It involves habitat management strategies to conserve and enhance beneficial organisms for effective pest control [89,93]. These habitat management practices support natural enemies by providing supplementary resources, including pollen, nectar, extrafloral nectar, alternative prey or hosts, and physical refuges [48,93]. Such outcomes can be achieved by modifying vegetation to promote biological control, regulate herbivorous insects, and minimize the use of plant protection chemicals [23]. 

Ecological engineering practices are generally compatible with rice production when properly designed [6,48,94]. Flower strips, hedgerows, and nectar-producing plants are typically planted along field edges, where they provide habitat and food resources for natural enemies without reducing the cultivated rice area [72,95]. These habitats provide nectar, shelter, and alternative resources for natural enemies [6,96]. During planting and routine crop management, these habitats require only occasional maintenance, such as trimming or replanting, and do not interfere with irrigation, fertilizer application, or pest monitoring [47,72]. For instance, a well-known ecological engineering program in Zhejiang Province, China, integrated sesame (*Sesamum indicum* L.) (Pedaliaceae) planting on rice bunds, reduced nitrogen fertilizer inputs, and avoided insecticide applications during the first 30 days after transplanting. These practices significantly increased the abundance of parasitoids and predatory arthropods, suppressed rice planthopper populations, reduced pesticide use, and maintained rice yields, demonstrating that ecological engineering can be successfully incorporated into routine rice production with minimal disruption to standard farming operations [72,95,97].

#### 3.3.1. Shelter for Natural Enemies

Establishing shelter for natural enemies of rice pests is vital for enhancing biological control, as it supports beneficial insects for overwintering, minimizes insecticide use, and facilitates rapid recolonization [48]. Shelter habitats provide semi-permanent environments with favorable biotic and abiotic conditions for overwintering, aestivation, reproduction, and protection from disturbances associated with agricultural practices such as plowing, spraying, and harvesting [98]. In addition, they enhance the abundance and diversity of natural enemies by providing favorable microclimates, alternative prey or hosts, non-prey food resources, and protection from intraguild predators and pesticide exposure [21,48]. Natural shelters can be provided by specific plant structures, plant groups, or plant litter that protect natural enemies [21,28,99] (Figure 2c). Among the most prevalent types of shelters are beetle bankers, shelterbelts, hedgerows, flower strips, intercropping, and the selective conservation of arable weeds [21] (Figure 1 and Figure 2). Retaining rice straw in rice fields (Figure 1a) and planting green manure crops such as Chinese milkvetch (*Astragalus sinicus* L.) (Fabaceae) (Figure 1e) after the rice harvest could provide habitats and shelter for native natural predators to overwinter [100]. Other ways of providing shelter include incorporating rice straw into the field (Figure 1d), maintaining Graminaceous plants around rice fields, intercropping with Manchurian wild rice (*Zizania latifolia* Griseb.) Turcz. ex-Stapf (Poaceae) (Figure 2b) and neighboring vegetable crops (Figure 2a), which increase natural enemies around and within the rice field [48]. These practices can suppress pest populations, minimizing the need for pesticides and promoting sustainable pest management [48,97].

Most shelter-based habitat management practices can be incorporated into rice production with minimal additional cost by utilizing existing non-cropped areas and routine field operations [6,48]. Flower strips, hedgerows, and shelterbelts can be established on field bunds, irrigation canals, and field margins, thereby avoiding competition with rice cultivation while providing shelter and floral resources for natural enemies [6,97]. Likewise, rice straw can be retained or incorporated after harvest rather than burned or removed, and green manure crops such as *Astragalus sinicus* can be grown during the fallow period to provide overwintering habitat for beneficial arthropods and improve soil fertility [48,101,102]. Conserving naturally occurring grasses and compatible vegetation around field edges requires little additional investment, and integrating *Zizania latifolia* or vegetable crops into suitable production systems can provide both ecological and economic benefits by enhancing natural enemy populations and strengthening biological control [6,12,48] (Figure 2a,b). Because these practices rely primarily on locally available resources and are incorporated into routine crop management, they require relatively low establishment costs while reducing pesticide dependence, conserving biodiversity, and supporting long-term biological control. 

Providing structured shelter within ecological engineering frameworks is essential for conserving natural enemies, protecting them from chemical disturbances, and facilitating winter survival [48]. Post-harvest fields present severe ecological disruptions; thus, specific agronomic practices must be deployed. Figure 1 details the cyclical management of overwintering shelters. The vibrant crop vegetation before harvest (Figure 1a) shifts to a bare landscape after harvest (Figure 1b), which lacks protective structures [6,96]. To counteract this, maintaining piles of retained rice straw within the field boundaries (Figure 1c) provides an insulating thermal layer that protects native predatory beetles and spiders from freezing temperatures [103]. Similarly, incorporating chopped rice straw directly into the soil surface (Figure 1d) preserves interstitial spaces that serve as micro-refuges [103]. Post-harvest cultivation of green manure crops, such as *A. sinicus* (Figure 1e), provides alternate vegetative structure and nectar resources, supporting parasitoid survival through winter transitions [6,94,104].

In-season spatial modifications are further illustrated in Figure 2. Planting diverse vegetable crops directly adjacent to the primary rice plots (Figure 2a) expands local dietary resources. Intercropping rice, such as *Z. latifolia* (Figure 2b), increases canopy structural complexity and provides stable microclimates. Furthermore, establishing multi-strata natural shelterbelts consisting of perennial shrubs and grasses (Figure 2c) and sowing dedicated flower strips along field bunds (Figure 2d) offer vital non-prey food sources such as nectar, which greatly extend the lifespans of parasitoid wasps [25,48,96].

#### 3.3.2. Alternative Host for Natural Enemies 

The functional synchronization between pests and natural enemies is highly mediated by rice phenological stages, which progress through distinct phases: tillering → booting → heading → grain filling → ripening. During the early tillering phase, primary crop pests are scarce, creating a severe resource bottleneck for specialist parasitoids. Sowing non-crop banker plants solves this issue by supporting harmless alternative herbivores. For instance, functional plants such as *Z. latifolia* are used to maintain stable populations of the non-pest planthopper *Saccharosydne procerus* (Matsumura), thereby supporting natural enemy populations and enhancing biological control in rice ecosystems [98]. This non-pest herbivore serves as a critical alternative host for *Anagrus* egg parasitoids during early tillering, allowing the parasitoids to multiply and suppress BPH populations before they reach damaging levels during the booting and heading stages [48,105].

**Figure 3:**
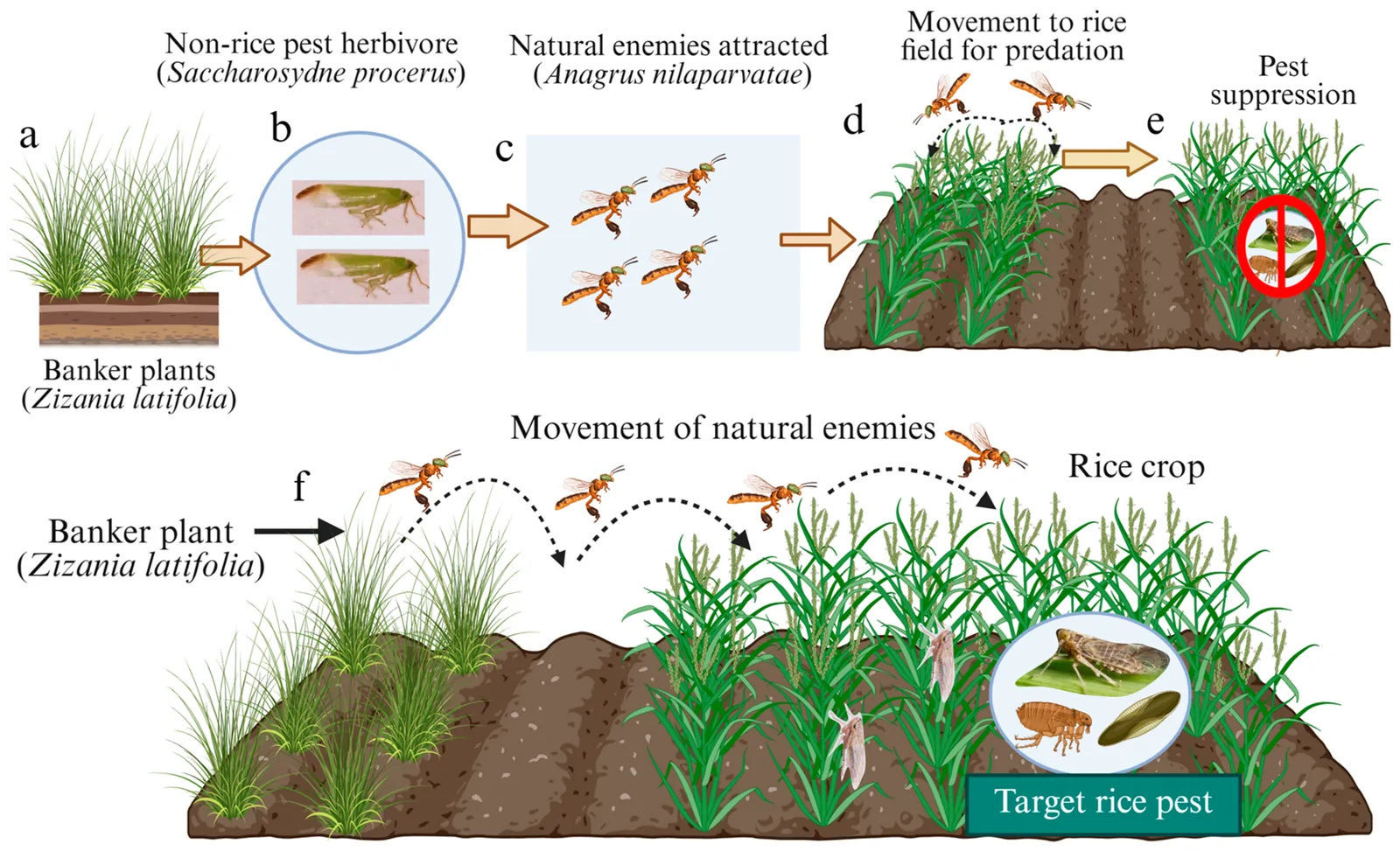
The banker plant system conserves natural enemies for sustainable pest management in rice. (**a**) banker plants (*Zizania latifolia*) serves as shelter and an alternative host/prey for beneficial insects; (**b**) the banker plant supports non-rice pest herbivores that thrive on them; (**c**) predators and parasitoids are attracted and sustained by these herbivores and the plant resources; (**d**) movement of natural enemies from the banker plants to the rice crop and attack target rice pests; (**e**) increased natural enemy populations suppress rice pests naturally; (**f**) natural enemies supported and maintained with the banker plant (*Zizania latifolia*) move to rice field suppress target rice pests such as planthoppers, leaf folders, and stem borers.

The banker plant system is a CBC method that involves nurturing a non-crop plant to support a non-pest herbivore, which attracts natural predators of crop pests [27,48]. The first successful banker plant system was developed in 1977, involving tomato as the banker plant, a parasitoid *Encarsia formosa* Gahan (Hymenoptera: Aphelinidae), and a whitefly pest *Trialeurodes vaporariorum* Westwood (Homoptera: Aleyrodidae) on tomatoes in greenhouses [106]. Banker plants provide essential food sources, such as shelter, pollen, and nectar, or various hosts or prey, fostering self-sustaining systems that promote the reproduction of beneficial organisms in agricultural environments to control a specific insect pest [98,107] (Figure 3). 

Ideally, the alternative host is a specialist of the banker plant to avoid harming other crops, and the natural enemies must be able to move across the field and target the main pest effectively [48]. Banker plants prevent the local extinction of natural enemies by providing alternative hosts or prey when pest populations are scarce, thereby sustaining and increasing beneficial arthropod populations [108,109]. In rice agroecosystems, predatory ladybird beetles (*Coccinella* spp.) and egg parasitoids (*Anagrus* and *Oligosita* spp.) are commonly associated with wild grasses along rice bunds, which provide prey during winter and the early rice-growing season when rice pests are rare [13,105]. Consequently, banker plants are established near crops to maintain natural enemy populations and enhance their biological control services throughout the cropping season [105] (Figure 3). 

Preserving and utilizing natural predators in rice fields efficiently controls RPHs [48]. Weeds provide shelter for *A. nilaparvatae*, a key egg parasitoid, while delphacids act as alternative hosts [110]. Examples of banker plant systems employed in rice production are *Z. latifolia*–*Saccharosydne procerus*–*A. nilaparvatae* (ZSA) and *Leersia* (*Leersia sayanuka* Ohwi) (Poacea*e*)–*Nilaparvata muiri*–*A. nilaparvatae,* and *Typhus chinensis* (LNA and T) [48]. Laboratory research indicated that the BPH could not complete its life cycle on *L. sayanuka*, while *N. muiri* was also unable to develop on rice [48]. Therefore, the cultivation of *L. sayanuka* poses no risk of serving as an alternative host for the rice pest BPH, as field studies show that BPH densities are significantly lower in rice fields integrated with this banker plant than in control fields without it [48,105]. The ZSA system involves intercropping rice with the aquatic vegetable *Z. latifolia* (Figure 3). 

The green slender planthopper (GSPH) is the main pest of *Z. latifolia* and provides winter food for the egg parasitoid *A. nilaparvatae*, which it shares with RPHs and leafhoppers [48,111]. Guo [112] found that rice fields with *L. sayanuka* spaced 5 m apart alongside *S. indicum* had higher populations of *A. nilaparvatae* during the tillering stage than fields with only *L. sayanuka* or control fields. Their study also indicated significant differences in parasitism rates, which were highest in fields featuring the 5 m combination of *L. sayanuka* and *S. indicum*. Additionally, spider populations were notably higher in this treatment than in others. During the rice tillering stage, fields planted with a combination of *L. sayanuka* and *S. indicum* in strips measuring either 50 cm × 5 m or 50 cm × 1 m exhibited significantly lower RPH populations than the control fields [112]. However, protection levels against natural enemies vary among host plants, emphasizing the need for research on optimal plant–host combinations for conservation biological control [108]. If chosen wisely, plants like *Zizania* can serve a dual role by offering shelter for various natural enemy taxa while also supporting hosts for more specialized parasitoid species [48]. These findings suggest that carefully selected non-crop plants can strengthen conservation biological control by providing habitat and alternative hosts for natural enemies.

Functional plants are essential for supporting natural enemies by conserving predators and recruiting parasites, stabilizing habitats, and providing alternative foods that enhance their abundance and persistence [92,113]. Plant functional types are defined as sets of species that exhibit similar responses to environmental conditions and have comparable effects on ecosystem functioning [114,115]. For instance, woody plants such as Chinese chaste tree (*Vitex negundo* L.) (Lamiaceae) and peach (*Prunus persica* L.) (Rosaceae) maintain predator populations year-round through complementary phenology; *V. negundo* supplies alternative prey during the wheat–rice transition, and its dense structure prevents seasonal declines during fallow periods [92]. 

Moreover, functional plants play a vital role in supporting parasitoids by providing essential nutrients and chemical cues [92]. Adult parasitoids rely on sugar sources to maximize lifespan and reproduction [18]; for example, Basil (*Ocimum basilicum* L.) (Lamiaceae) flowers have been shown to enhance parasitism rates by offering consistent floral resources [116]. Beyond nutrition, functional plants recruit parasitoids through chemical signals; for instance, Mung bean (*Vigna radiata* (L.) R. Wilczek) (Fabaceae) emits C6 alkene and alcohol volatiles that boost egg parasitoid populations in corn systems [117]. The combined effects of these functions make functional plants effective and sustainable biological control agents that conserve and enhance natural enemy diversity [6,118].

#### 3.3.3. Floral Resources for Natural Enemies 

Flower strips have been incorporated into agricultural practices since the 1990s, particularly in Europe, as part of agri-environmental schemes [119,120]. These strips provide multiple benefits, such as habitat for beneficial insects, enhanced pollination services, and improved pest control [119,121] (Figure 2d). Ideally, flower strips should feature native, perennial species that bloom throughout the growing season to support key beneficial insects and can comprise a mix of annuals and perennials [119,122]. They have been effectively used in perennial crops and greenhouses, where non-crop areas allow for implementation, such as between tree rows [119,123,124,125]. However, integrating perennial strips into annual field crops poses challenges due to disturbances from plowing, pesticide use, land allocation issues, and large field sizes [119,123,124]. While field margins can be utilized, they may provide limited resources for natural enemies. The effectiveness of flower strips in open landscapes is influenced by their size, quality, and the selection of flowering species [119,126]. 

The abundance of natural enemies increases markedly with the species richness of flower strips [127] (Figure 2d). Greater plant diversity enhances habitat complexity, providing a wider variety of pollen, nectar, and alternative prey, which supports more arthropod natural enemies [119]. While species richness is critical, selecting flower species with functional traits that benefit both specialist and generalist natural enemies is essential [119,128]. Flower strip quality, determined by the suitability of floral resources for natural enemies, also influences effectiveness. For example, He et al. assessed flower strip quality by scoring species based on their effects on predator longevity and demonstrated that higher-quality flower strips supported greater abundances of natural enemies [129]. Providing suitable host plants can enhance predator longevity and disease resistance, further boosting their populations [129,130]. In contrast, single-species flower strips provide a narrower range of resources, characterized by shorter flowering periods and lower structural diversity, thereby supporting fewer natural enemies for a shorter duration [119,131]. However, certain single-species strips, like buckwheat (*Fagopyrum esculentum* Moench) (Polygonaceae) or sweet alyssum (*Lobularia maritima* (L.) Desv.) (Brassicaceae), can perform well due to their extended flowering periods and high value for predators [119,129,132]. 

Establishing nectar-producing flower strips or patches on rice bunds (levees) can increase the abundance of natural enemies and help reduce pest incidence [25,72,133]. Key predators and parasitoids are often abundant in rice fields near vegetation strips [25], potentially reducing the need for insecticide applications and improving rice yields [72,133]. Access to pollen and nectar is crucial for these beneficial organisms; for example, predators like syrphids can travel longer distances to find these essential resources [119] (Figure 2d). Epigeal predators thrive in complex habitats that offer plenty of overwintering refuges, although their ability to colonize new areas may be limited [134]. Combining resistant rice varieties with vegetation strips can amplify the advantages of both strategies. For instance, vegetation strips paired with resistant rice varieties can further reduce populations of *S. furcifera* and green leafhopper (*Nephotettix virescens* Distant) (Cicadellidae) by enhancing predator and parasitoid diversity near the bunds [25]. Greater arthropod diversity, including herbivores, near vegetation strips is expected to stabilize communities by broadening species interactions, including competition [25]. 

Generally, predators and parasitoids typically disperse from flower strips into adjacent rice fields, where their biological control effects are greatest near the habitat and gradually decline with increasing distance [6,95,98]. The extent of dispersal varies among species and is influenced by landscape structure, habitat connectivity, and the rice growth stage. Egg parasitoids, such as *Anagrus* spp. and *Oligosita* spp., actively disperse up to 10–30 m from flowering strips while searching for host insects [134]. Likewise, generalist predators, including spiders and ladybird beetles, move from flower strips into rice crops, although their abundance generally decreases with increasing distance from the habitat [134]. In contrast, highly mobile flying predators, such as dragonflies and lacewings, can readily colonize entire rice fields and are therefore less dependent on the proximity of flowering strips [25].

Floral resources enhance the effectiveness of natural enemies by attracting them, prolonging their lifespan, and boosting their reproductive capacity [135]. Therefore, planting appropriate flowering species in non-crop areas around paddy bunds can enhance the abundance and diversity of key natural enemies, ultimately helping to reduce pest populations [48]. Floral resources lead to higher abundance and reproduction rates of natural enemies, especially in rice systems, resulting in lower pest populations, increased yields, reduced pesticide use, and greater economic returns [48,135,136]. Olfactometric tests have shown that flowers from marigold, cowpea, and sesame attract significantly higher numbers of key predators—such as *Micraspis discolor*, *Ophionea nigrofasciata*, and *C. lividipennis*—as well as parasitoids like *T. chilonis* and *Trichogramma japonicum* Ashmead (Trichogrammatidae), compared to control treatments [93]. Consistent with these findings, field studies have also recorded higher populations of natural enemies, including coccinellids, ground beetles, rove beetles, mirid bugs, damselflies, and spiders, in plots containing flowering plants than in control plots [93]. This attraction is likely driven by volatile compounds emitted from the flowers. Sesame is particularly valued in ecological engineering for rice pest management due to its dual income benefits and its inclusion in China's nationally recommended sustainable pest control strategies [48]. 

Sowing flowering strips on field bunds provides vital nectar and pollen that directly improve natural enemy performance. Recent meta-analyses by Jachowicz and Sigsgaard [119] provide crucial quantitative effect sizes for these interventions: highly diverse flower strips comprising two or more sown plant species increase natural enemy abundance by an average of 70% (±24.7% SE) within adjacent crops. Crucially, their modeling reveals a linear benefit where each additional plant species incorporated into the strip increases natural enemy abundance by approximately 3.5–4.1%. This quantitative enhancement occurs because high plant richness ensures overlapping blooming periods and varied floral architectures, which accommodate both generalist ladybird beetles and short-tongued parasitoid wasps.

## 4. Integrated Pest Management 

**Figure 4:**
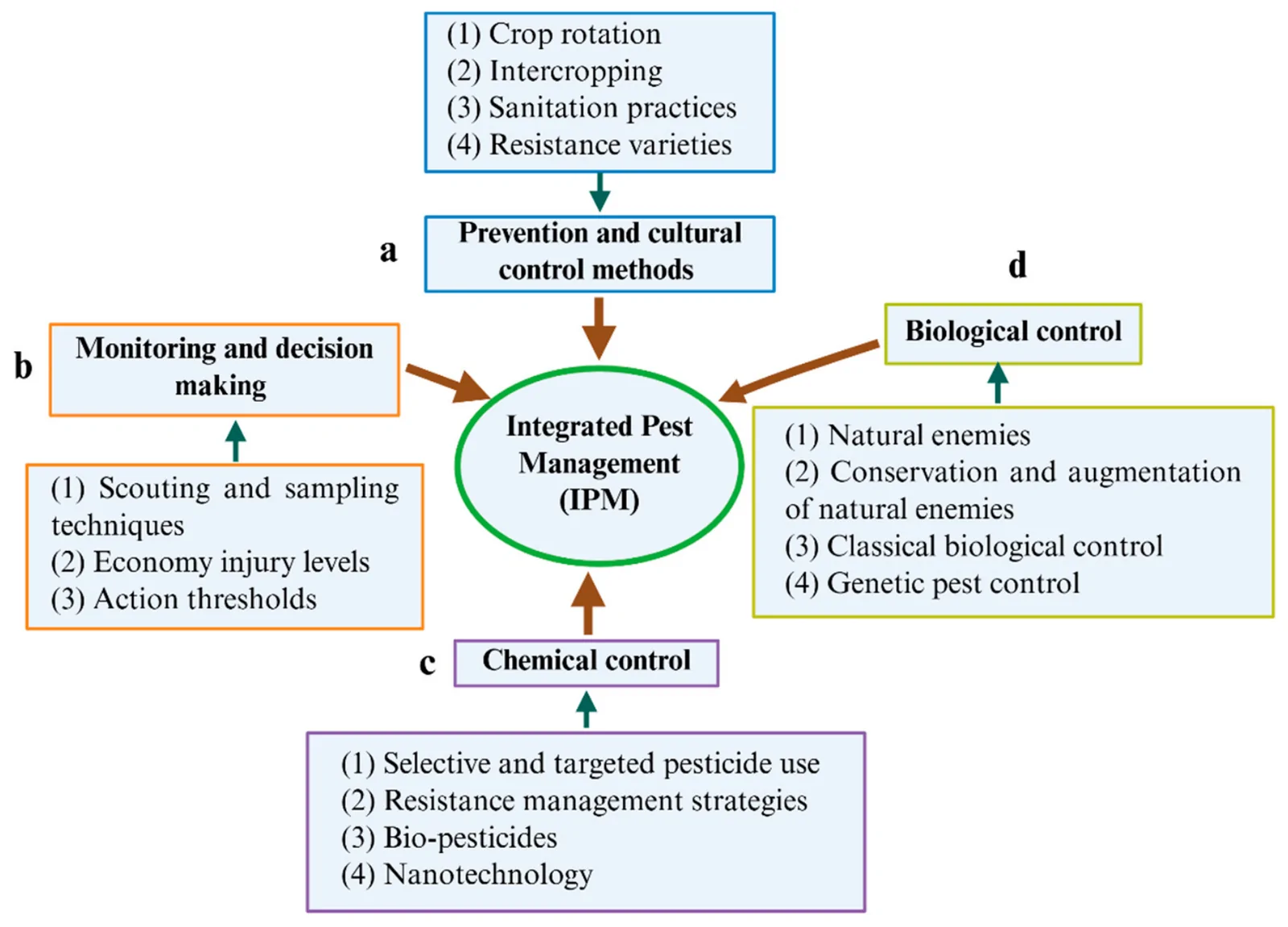
Essential elements of IPM involve: (**a**) prevention and cultural practices such as crop rotation and the use of resistant varieties; (**b**) monitoring through pest scouting and evaluating economic injury thresholds; (**c**) chemical controls that include targeted pesticide application and strategies for managing pesticide resistance; (**d**) biological control strategies for sustainable pest management.

IPM is an environmentally sustainable approach that employs a variety of pest control strategies to keep pest populations below economically damaging levels while reducing risks to human health and the environment [137]. Instead of relying solely on reactive pesticide applications, IPM focuses on a coordinated integration of cultural, biological, physical, and chemical control methods [138,139] (Figure 4). This approach prioritizes preventive measures, continuous monitoring, and decision-making based on established economic thresholds, ensuring effective and sustainable pest management.

Key principles of IPM include preventing pest outbreaks through cultural practices such as crop rotation, sanitation, intercropping, and cultivating resistant crop varieties, which help create unfavorable conditions for pest development [140,141] (Figure 4a). Regular monitoring through scouting and the establishment of action thresholds allows farmers to evaluate pest populations and determine the right timing for intervention [137,141] (Figure 4b). Biological control strategies rely on natural enemies, such as predators, parasitoids, and other beneficial organisms, to regulate pest populations and provide sustainable pest suppression [142] (Figure 4d). If other methods prove inadequate, chemical control measures, such as biopesticides and selectively targeted pesticides, are applied judiciously to minimize negative environmental impacts [137] (Figure 4c). By integrating these diverse strategies, IPM offers an effective, economically viable, and ecologically sound approach to pest management in agricultural systems.

### Conceptual Relationship Between Integrated Pest Management, Conservation Biological Control, Habitat Management, and Ecological Engineering

Integrated Pest Management (IPM) offers a sustainable crop protection strategy that integrates biological, cultural, physical, and chemical methods to keep pest populations below economic injury levels while protecting human health and the environment [139]. A vital aspect of IPM is biological control, particularly conservation biological control (CBC), which focuses on preserving and enhancing natural predators and parasitoids through farming practices that support their survival, reproduction, and effectiveness in suppressing pest populations [6,96]. 

Habitat management plays a key role in CBC by optimizing crop and non-crop areas to provide resources such as nectar, pollen, and shelter for these beneficial organisms [6,96,143]. Ecological engineering builds on habitat management by applying ecological principles to create diverse agroecosystems that enhance biodiversity and pest control services [6]. Strategies such as flowering margins, banker plant systems, cover crops, and vegetated bunds increase the abundance and effectiveness of natural enemies, thereby reducing pest outbreaks [6,144]. Therefore, habitat management and ecological engineering are complementary strategies that support conservation biological control, which in turn constitutes an important biological control component within the broader integrated pest management framework for sustainable crop insect pest management.

## 5. Case Studies and Evidence from Biocontrol Systems

Although the evidence base remains geographically uneven, several representative studies illustrate how habitat management can improve pest suppression when interventions are aligned with local ecological conditions and farming systems.

### 5.1. Landscape Heterogeneity and Natural Pest Suppression in Bangladesh

A two-year study in the Patuakhali and Satkhira regions of Bangladesh examined how landscape composition and configuration affect the biological control of the BPH across multiple spatial scales (Table 2). They found that ladybird beetle populations were notably influenced by landscape structure, thriving in areas with fallow lands and vegetated road edges, highlighting these habitats as crucial refuges and resources for predators [13]. In contrast, spider abundance was less affected by landscape variables and primarily hindered by the rice phenological stage. The study also introduced a biocontrol service index, which showed a positive correlation with landscape diversity and a negative correlation with pest density and yield loss. This suggests that more heterogeneous landscapes enhance biological control services and boost crop productivity. 

### 5.2. Ecological Engineering in Zhejiang, China

Large-scale ecological engineering initiatives in Zhejiang Province incorporated the use of flowering plants, reduced insecticide application, and minimized nitrogen inputs across rice farms (Table 2). Following this initiative, similar field studies were conducted on various farms in Zhejiang, including Ningbo, Xiaoshan, Lishui, Wenling, and Wenzhou, with each study covering more than 10 hectares. Field surveys demonstrated that ecological engineering increased the abundance of *Anagrus* spp. and various invertebrate predators, including damselflies such as *Ischnura senegalensis* Rambur and *Agriocnemis femina*, by more than fourfold compared with conventionally managed farms [81,97]. Furthermore, this ecological engineering strategy doubled the abundance of RPH egg parasitoids and reduced RPH populations by more than fivefold during the tillering and grain-filling stages relative to conventional management practices. 

### 5.3. Landscape Composition and Parasitoid Responses in Stem-Borer Systems

Studies of rice stem-borer systems in subtropical landscapes have shown that landscape composition influences both pest infestation and parasitoid performance [145] (Table 2). However, specialist parasitoids do not always benefit from increased non-crop habitat because host availability, dispersal ability, and species-specific life-history traits mediate their responses. For example, the specialist parasitoid *C. chilonis* responded negatively to increasing non-crop habitat, whereas generalist parasitoids such as *Eriborus sinicus* and *Microgaster russata* responded positively, highlighting the importance of considering functional traits when interpreting landscape-diversification effects on biological control [145]. This case illustrates why life-history traits must be considered when generalizing from landscape-diversification studies.

**Table 2:** Representative case studies illustrating habitat-based biological control in rice systems.

Case Study	System	Intervention	Main Target Pest(s)	Main Outcome	References
Bangladesh’s heterogeneous rice landscapes	Smallholder rice mosaic	Fallow land and vegetated road edges	Brown planthopper	Higher predator abundance and stronger natural pest suppression	[13]
Zhejiang ecological engineering program	Operational rice farms	Flowering plants, reduced insecticide, lower nitrogen	Planthoppers	Higher parasitoid abundance and lower pest pressure	[81,97]
Subtropical stem-borer systems	Landscape-varied rice production	Landscape-level habitat heterogeneity	Stem borers	Species-specific parasitoid responses to landscape composition	[145]

## 6. Challenges and Future Research Agenda

Several challenges continue to hinder the scientific understanding and practical implementation of habitat management in rice cultivation. Firstly, while many studies quantify changes in the abundance of natural enemies, they often fail to establish robust connections between these changes and outcomes such as pest suppression, yield stability, or overall farm profitability. Secondly, much of the existing literature is dominated by short-term experiments, which complicates the assessment of persistence over time, variations in climate, and evolving pest dynamics. Thirdly, the current evidence base is largely focused on Asia, neglecting the unique ecological and socio-economic contexts present in rice systems across Africa and South America [6,39,146]. 

To address these gaps, future research should focus on four key areas. One priority should be to improve testing of mechanisms related to plant traits, volatile cues, natural enemy nutrition, and movement ecology. Another important direction is to design experiments that account for both field-level interventions and landscape contexts. Additionally, there is a need for standardized reporting of effect sizes for natural enemy abundance, parasitism, predation, pesticide reduction, and yield outcomes. Lastly, it is essential to integrate socio-economic analyses that consider factors such as labor requirements, profitability, farmer perceptions, and policy incentives related to ecological engineering [141,147]. 

The urgency of addressing these issues is amplified by climate change. Warming temperatures, altered rainfall patterns, and shifting crop and insect phenology are expected to reshape pest pressures and the performance of natural enemies. Therefore, habitat management strategies must be resilient to climate change while maintaining biological functionality [148]. Future ecological engineering efforts should prioritize incorporating locally adapted plant mixtures that provide multiple ecosystem services, including nectar resources, structural refuge, and enhanced support for biodiversity.

## 7. Conclusions and Prospects

Habitat management holds significant promise for enhancing conservation biological control within rice ecosystems, but its effectiveness hinges on ecological compatibility rather than mere diversification. Key strategies include planting nectar-rich flowering species along field bunds, creating banker plant refuges, preserving fallow areas, and maintaining diverse vegetation strips adjacent to rice paddies [48,94,98]. These enhancements provide essential resources—such as shelter, nectar, and alternative prey—for natural enemies, including predators and parasitoids that target major rice pests like planthoppers and stem borers [48,149,150].

Recent evidence indicates that landscape composition and configuration, collectively referred to as landscape heterogeneity, play a critical role in determining the abundance and effectiveness of these natural enemies [13,151,152]. Research shows that diverse habitats, like road edges and fallow lands, can support higher populations of beneficial species, such as ladybird beetles and spiders, which are instrumental in controlling BPH populations and reducing yield losses [13,153]. Moreover, studies suggest a positive correlation between landscape diversity and pest suppression, implying that maintaining heterogeneous, fragmented habitats can reduce the need for preventive insecticide applications [13].

By blending landscape-level diversity with targeted on-field habitat management, ecological engineering offers a scalable and cost-effective approach to enhancing yields, diminishing chemical inputs, conserving biodiversity, and promoting the sustainable intensification of rice production. This strategic habitat manipulation represents a significant shift in global rice protection, encouraging farmers to substitute broad-spectrum chemical treatments with structured non-crop resources. Such a transition not only stabilizes top-down biocontrol services but also ensures high yields and safeguards agricultural biodiversity.

Looking ahead, future research should aim to quantify the specific impacts of various habitat modifications on pest suppression and natural enemy abundance, while also exploring climate-resilient plant species. By developing adaptive strategies to accommodate shifting environmental conditions, we can improve the effectiveness of habitat management in rice agroecosystems and bolster long-term sustainability in global agriculture.

## Data Availability

No new data were created or analyzed in this study. Data sharing does not apply to this article.

## Abbreviations

The following abbreviations are used in this manuscript:

BPH	Brown planthopper
CBC	Conservation biological control
GSPH	Green slender planthopper
IPM	Integrated pest management
RPHs	Rice planthoppers
RYMV	Rice Yellow Mottle Virus
WBPH	White-backed planthopper
VOCs	Volatile organic compounds

## References

[1] Mohidem N.A., Hashim N., Shamsudin R., Che Man H. (2022). Rice for food security: Revisiting its production, diversity, rice milling process and nutrient content. Agriculture.

[2] Hang S., Wang Q., Wang Y., Xiang H. (2024). Evolution of rice cultivar performance across China: A multi-dimensional study on yield and agronomic characteristics over three decades. Agronomy.

[3] Xu J. (2025). From grain to diversity: The role of rice in shaping the dietary and cultural fabric of eastern China. Int. J. Gastron. Food Sci..

[4] Rao A., Wani S., Ramesha M., Ladha J. (2017). Rice production systems. Rice Production Worldwide.

[5] Joshi E., Kumar D., Lal B., Nepalia V., Gautam P., Vyas A.K. (2013). Management of direct-seeded rice for enhanced resource—Use efficiency. Plant Knowl. J..

[6] Gurr G.M., Wratten S.D., Landis D.A., You M. (2017). Habitat management to suppress pest populations: Progress and prospects. Annu. Rev. Entomol..

[7] Fukai S., Wade L.J., Sadras V.O., Calderini D.F. (2021). Chapter 2—Rice. Crop Physiology Case Histories for Major Crops.

[8] Huang S., Wang L., Liu L., Fu Q., Zhu D. (2014). Nonchemical pest control in China rice: A review. Agron. Sustain. Dev..

[9] Yang Q., Jaworski C.C., Wen Z., Desneux N., Ouyang F., Dai X., Wang L., Jia J., Zheng H. (2025). Crop heterogeneity may not enhance biological control of rice pests in landscapes rich in semi-natural habitats. Agric. Ecosyst. Environ..

[10] Zhang H., He B., Xing J., Lu M. (2022). Spatial and temporal patterns of rice planthopper populations in South and Southwest China. Comput. Electron. Agric..

[11] Pegalepo E., Bocco R., Onaga G., Nwilene F., Tamò M., Togola A., Katiyar S.K. (2025). Sustainable insect pest management options for rice production in Sub-Saharan Africa. Insects.

[12] Zhu P., Liang R., Qin Y., Xu H., Zou Y., Johnson A.C., Zhang F., Gurr G.M., Lu Z. (2023). Extrafloral and floral nectar promote biocontrol services provided by parasitoid wasps to rice crops. Entomol. Gen..

[13] Ali M.P., Clemente-Orta G., Kabir M.M.M., Haque S.S., Biswas M., Landis D.A. (2023). Landscape structure influences natural pest suppression in a rice agroecosystem. Sci. Rep..

[14] Nchu F. (2024). Sustainable biological control of pests: The way forward. Appl. Sci..

[15] Brzozowski L., Mazourek M. (2018). A sustainable agricultural future relies on the transition to organic agroecological pest management. Sustainability.

[16] Valean A.M., Suciu L., Tărău A., Șopterean L., Russu F., Șimon A., Chețan F., Tritean N. (2025). Impact of shelterbelts on the diversity and dynamics of natural enemies in wheat agroecosystems. Agronomy.

[17] Abdul Qayyum M., Saeed S., Naeem-Ullah U., Wajid M., Siddique A., Matloob A., Shahid R., Ur Rehman Zia H.U., Bilal H., Ramzan M., Ansari M.-u.-R. (2021). Insect Pest Complex of Wheat Crop. Current Trends in Wheat Research.

[18] Baker B.P., Green T.A., Loker A.J. (2020). Biological control and integrated pest management in organic and conventional systems. Bio. Control.

[19] McCravy K.W. (2018). A review of sampling and monitoring methods for beneficial arthropods in agroecosystems. Insects.

[20] Rusch A., Bommarco R., Ekbom B. (2017). Conservation biological control in agricultural landscapes. Adv. Bot. Res..

[21] Gontijo L.M. (2019). Engineering natural enemy shelters to enhance conservation biological control in field crops. Bio. Control.

[22] Gong S., Zhu Y., Fu D., Bianchi F.J., van Der Werf W., Hodgson J.A., Xiao H., Zou Y. (2024). Land consolidation impacts the abundance and richness of natural enemies but not pests in smallholder rice systems. J. Appl. Ecol..

[23] Heong K.L., Lu Z.-X., Chien H.V., Escalada M., Settele J., Zhu Z.R., Cheng J.A. (2021). Ecological engineering for rice insect pest management: The need to communicate widely, improve farmers’ ecological literacy, and policy reforms to sustain adoption. Agronomy.

[24] Rosida N., Ibrahim E., Senoaji W., Sidik E.A., Mugiasih A., Hutasoit R.T., Praptana R.H., Sudewi S., Nasruddin A. (2025). Harnessing flowering bund plants through ecological engineering to improve biological control of tungro virus vectors in Indonesian rice fields agro-ecosystem. Int. J. Food Sci..

[25] Ali M.P., Bari M.N., Haque S.S., Kabir M.M.M., Afrin S., Nowrin F., Islam M.S., Landis D.A. (2019). Establishing next-generation pest control services in rice fields: Eco-agriculture. Sci. Rep..

[26] Wang J., Yang Y., Li Y., Jin Z., Desneux N., Han P., Wang S., Li S. (2022). Direct and indirect effects of banker plants on population establishment of *Harmonia axyridis* and aphid control on pepper crop. Front. Plant Sci..

[27] Frank S.D. (2010). Biological control of arthropod pests using banker plant systems: Past progress and future directions. Bio. Control.

[28] Landis D.A. (2017). Designing agricultural landscapes for biodiversity-based ecosystem services. Basic. Appl. Ecol..

[29] Del-Val E., Ramírez E., Astier M. (2021). Comparison of arthropod communities between high and low input maize farms in Mexico. CABI Agric. Biosci..

[30] Elhamalawy O., Bakr A., Eissa F. (2024). Impact of pesticides on non-target invertebrates in agricultural ecosystems. Pestic. Biochem. Physiol..

[31] Michalko R., Pekár S., Entling M.H. (2019). An updated perspective on spiders as generalist predators in biological control. Oecologia.

[32] Jarpla M., Kumari P., Pawar P., Reddy N., Bhargavi C., Samreen M., Aniket E. (2024). A review on the role of beneficial insects in sustainable crop production systems. J. Exp. Agric. Int..

[33] Smith R.F., Bosch R.v.d., Hagen K.S., Stern V.M. (1959). The integration of chemical and biological control of the spotted alfalfa aphid: The integrated control concept. Hilgardia.

[34] Bottrell D.G., Schoenly K.G. (2012). Resurrecting the ghost of green revolutions past: The brown planthopper as a recurring threat to high-yielding rice production in tropical Asia. J. Asia-Pac. Entomol..

[35] Pedigo L.P. (1996). Entomology and Pest Management.

[36] Ratto F., Bruce T., Chipabika G., Mwamakamba S., Mkandawire R., Khan Z., Mkindi A., Pittchar J., Sallu S.M., Whitfield S. (2022). Biological control interventions reduce pest abundance and crop damage while maintaining natural enemies in sub-Saharan Africa: A meta-analysis. Proc. Biol. Sci..

[37] Heong K.L., Hardy B. (2009). Planthoppers: New Threats to the Sustainability of Intensive rice Production Systems in Asia.

[38] Pathak M.D., Khan Z.R. (1994). Insect Pests of Rice.

[39] Conde S., Catarino S., Ferreira S., Temudo M.P., Monteiro F. (2025). Rice pests and diseases around the world: Literature-based assessment with emphasis on Africa and Asia. Agriculture.

[40] Wilson B.E., Villegas J., Stout M., Landry K. (2021). Relative yield loss from stem borers (Lepidoptera: Crambidae) and rice water weevil (Coleoptera: Curculionidae) in rice. J. Econ. Entomol..

[41] Chen B., Chen Y., Chen H., Liang Z., Chen J., Wu R., Zhang T., Zhou G., Yang X. (2023). Identification, characterization and prevalence in southern China of a new *Recilia iflavirus* in the leafhopper *Recilia dorsalis* (Hemiptera: Cicadellidae). Virus Res..

[42] Horgan F.G., Crisol E. (2013). Hybrid rice and insect herbivores in Asia. Ent. Exp. appl..

[43] Hsu G.C., Ou J.A., Ni M.H., Lin Z.H., Ho C.K. (2025). Generalist predators function as pest specialists: Examining diet composition of spiders and ladybeetles across rice crop stages. Appl. Ecol..

[44] Chaitanya M., Pavan J., Vireesha P., Nekkanti A. (2024). Natural enemies as guardians of crop ecosystem with special emphasis on rice and cotton. J. Exp. Agric. Int..

[45] Shi L., Liu D., Qiu L., Jiang Z., Zhan Z. (2022). Evaluation of the parasitism capacity of a thelytoky egg parasitoid on a serious rice pest, *Nilaparvata lugens* (Stål). Animals.

[46] Nakanishi K., Yokomizo H., Hayashi T.I. (2018). Were the sharp declines of dragonfly populations in the 1990s in Japan caused by fipronil and imidacloprid? An analysis of Hill’s causality for the case of *Sympetrum frequens*. Environ. Sci. Pollut. Res..

[47] Gurr G.M., Liu J., Read D.M.Y., Catindig J.L.A., Cheng J.A., Lan L.P., Heong K.L. (2011). Parasitoids of Asian rice planthopper (Hemiptera: Delphacidae) pests and prospects for enhancing biological control by ecological engineering. Ann. Appl. Biol..

[48] Zhu P., Zheng X., Johnson A.C., Chen G., Xu H., Zhang F., Yao X., Heong K., Lu Z., Gurr G.M. (2022). Ecological engineering for rice pest suppression in China. A review. Agron. Sustain. Dev..

[49] Rosenheim J.A. (1998). Higher-order predators and the regulation of insect herbivore populations. Annu. Rev. Entomol..

[50] Way M.J., Heong K.L. (1994). The role of biodiversity in the dynamics and management of insect pests of tropical irrigated rice—A review. Bull. Entomol. Res..

[51] Joseph A.R., Premila K. (2016). A study on the richness of spider fauna in rice ecosystem. J. Entomol. Zool. Stud..

[52] Guo J., Fu X., Zhao S., Shen X., Wyckhuys K.A., Wu K. (2020). Long-term shifts in abundance of (migratory) crop-feeding and beneficial insect species in northeastern Asia. J. Pest Sci..

[53] Holland J.M., Bianchi F.J., Entling M.H., Moonen A.C., Smith B.M., Jeanneret P. (2016). Structure, function and management of semi-natural habitats for conservation biological control: A review of European studies. Pest. Manag. Sci..

[54] Shields M.W., Johnson A.C., Pandey S., Cullen R., González- Chang M., Wratten S.D., Gurr G.M. (2019). History, current situation, and challenges for conservation biological control. Bio. Control.

[55] Hassan K., Pervin M., Mondal F., Mala M. (2016). Habitat management: A key option to enhance natural enemies of crop pest. Univers. J. Plant Sci..

[56] Dainese M., Montecchiari S., Sitzia T., Sigura M., Marini L. (2017). High cover of hedgerows in the landscape supports multiple ecosystem services in Mediterranean cereal fields. J. Appl. Ecol..

[57] Beers E.H., Mills N.J., Shearer P.W., Horton D.R., Milickzy E.R., Amarasekare K.G., Gontijo L.M. (2016). Nontarget effects of orchard pesticides on natural enemies: Lessons from the field and laboratory. Bio. Control.

[58] Jacob H.S., Evans E.W. (2000). Influence of carbohydrate foods and mating on longevity of the parasitoid *Bathyplectes curculionis* (Hymenoptera: Ichneumonidae). Environ. Entomol..

[59] Wackers F.L. (1999). Gustatory response by the Hymenopteran parasitoid *Cotesia glomerata* to a range of nectar and honeydew sugars. J. Chem. Ecol..

[60] Mala M., Mollah M.M.I., Baishnab M. (2020). Importance of intercropping for biodiversity conservation. J. Sci. Technol. Environ. Inform..

[61] Tooker J.F., Frank S.D. (2012). Genotypically diverse cultivar mixtures for insect pest management and increased crop yields. J. Appl. Ecol..

[62] Perrin R., Phillips M. (1978). Some effects of mixed cropping on the population dynamics of insect pests. Ent. Exp. Appl..

[63] Altieri M.A. (1993). Ethnoscience and biodiversity: Key elements in the design of sustainable pest management systems for small farmers in developing countries. Agric. Ecosyst. Environ..

[64] Moreira B., Gonçalves A., Pinto L., Prieto M.A., Carocho M., Caleja C., Barros L. (2024). Intercropping systems: An opportunity for environmental conservation within nut production. Agriculture.

[65] Diyaolu C.O., Folarin I.O. (2024). The role of biodiversity in agricultural resilience: Protecting ecosystem services for sustainable food production. Int. J. Res. Publ. Rev..

[66] Lamzira R., El Fakhouri K., Boulamtat R., Kemal S.A., Oubayoucef A., Ramdani C., Meftah Kadmiri I., El Bouhssini M. (2025). Multifunctional roles of intercropping in the management of insect pests affecting pulse crops: A comprehensive bibliometric analysis. Front. Sustain. Food Syst..

[67] Markovic D., Seimandi-Corda G., Harizanova V., Stoeva A., Himanen S., Saussure S., Radonjic A., Đurić G., Lalićević I., Kheam S. (2025). Volatile-mediated plant interactions: An innovative approach to cultivar mixture selection for enhanced pest resilience. Front. Plant Sci..

[68] Remelli S., Gatti F., Tarocco P., Scotti C., Menta C. (2025). Below-ground biodiversity in agricultural systems: The role of crop-specific management in shaping soil arthropod communities. Agric. Ecosyst. Environ..

[69] Qiu T., Shi Y., Peñuelas J., Liu J., Cui Q., Sardans J., Zhou F., Xia L., Yan W., Zhao S. (2024). Optimizing cover crop practices as a sustainable solution for global agroecosystem services. Nat. Commun..

[70] Jaworski C.C., Thomine E., Rusch A., Lavoir A.-V., Wang S., Desneux N. (2023). Crop diversification to promote arthropod pest management: A review. Agric. Commun..

[71] Chaplin-Kramer R., O’Rourke M.E., Blitzer E.J., Kremen C. (2011). A meta-analysis of crop pest and natural enemy response to landscape complexity. Ecol. Lett..

[72] Gurr G.M., Lu Z., Zheng X., Xu H., Zhu P., Chen G., Yao X., Cheng J., Zhu Z., Catindig J.L. (2016). Multi-country evidence that crop diversification promotes ecological intensification of agriculture. Nat. Plants.

[73] Jeanneret P., Aviron S., Alignier A., Lavigne C., Helfenstein J., Herzog F., Kay S., Petit S. (2021). Agroecology landscapes. Landsc. Ecol..

[74] Tscharntke T., Karp D.S., Chaplin-Kramer R., Batáry P., DeClerck F., Gratton C., Hunt L., Ives A., Jonsson M., Larsen A. (2016). When natural habitat fails to enhance biological pest control–Five hypotheses. Biol. Conserv..

[75] Jaworski C.C., Thomine E., Rusch A., Lavoir A.-V., Xiu C., Ning D., Lu Y., Wang S., Desneux N. (2022). At which spatial scale does crop diversity enhance natural enemy populations and pest control? An experiment in a mosaic cropping system. Agronomy.

[76] Gagic V., Hänke S., Thies C., Scherber C., Tomanović Z., Tscharntke T. (2012). Agricultural intensification and cereal aphid–parasitoid–hyperparasitoid food webs: Network complexity, temporal variability and parasitism rates. Oecologia.

[77] Schneider G., Krauss J., Riedinger V., Holzschuh A., Steffan-Dewenter I. (2015). Biological pest control and yields depend on spatial and temporal crop cover dynamics. J. Appl. Ecol..

[78] Schneider G., Krauss J., Boetzl F.A., Fritze M.-A., Steffan-Dewenter I. (2016). Spillover from adjacent crop and forest habitats shapes carabid beetle assemblages in fragmented semi-natural grasslands. Oecologia.

[79] Laterza I., Dioli P., Tamburini G. (2023). Semi-natural habitats support populations of stink bug pests in agricultural landscapes. Agric. Ecosyst. Environ..

[80] Thomine E., Rusch A., Desneux N. (2023). Predators do not benefit from crop diversity but respond to configurational heterogeneity in wheat and cotton fields. Landsc. Ecol..

[81] Yang Q., Li Z., Ouyang F., Men X., Zhang K., Liu M., Guo W., Zhu C., Zhao W., Reddy G.V. (2022). Flower strips promote natural enemies, provide efficient aphid biocontrol, and reduce insecticide requirement in cotton crops. Entomol. Gen..

[82] Tscharntke T., Tylianakis J.M., Rand T.A., Didham R.K., Fahrig L., Batáry P., Bengtsson J., Clough Y., Crist T.O., Dormann C.F. (2012). Landscape moderation of biodiversity patterns and processes—Eight hypotheses. Biol. Rev..

[83] Tooker J.F., O’Neal M.E., Rodriguez-Saona C. (2020). Balancing disturbance and conservation in agroecosystems to improve biological control. Annu. Rev. Entomol..

[84] Dassou A.G., Tixier P. (2016). Response of pest control by generalist predators to local-scale plant diversity: A meta-analysis. Ecol. Evol..

[85] Rossetti M.R., Tscharntke T., Aguilar R., Batáry P. (2017). Responses of insect herbivores and herbivory to habitat fragmentation: A hierarchical meta-analysis. Ecol. Lett..

[86] Cardinale B.J., Harvey C.T., Gross K., Ives A.R. (2003). Biodiversity and biocontrol: Emergent impacts of a multi-enemy assemblage on pest suppression and crop yield in an agroecosystem. Ecol. Lett..

[87] Mkenda P.A., Ndakidemi P.A., Mbega E., Stevenson P.C., Arnold S.E.J., Gurr G.M., Belmain S.R. (2019). Multiple ecosystem services from field margin vegetation for ecological sustainability in agriculture: Scientific evidence and knowledge gaps. PeerJ.

[88] Wang C., Bian Z., Wang S., Liu X., Zhang Y. (2022). The effect of artificial field margins on epigeic arthropod functional groups within adjacent arable land of Northeast China. Land.

[89] Bommarco R. (2024). Ecological redesign of crop ecosystems for reliable crop protection. A review. Agron. Sustain. Dev..

[90] Evans A. (2005). Ecological engineering for pest management. Agric. Sci..

[91] Huss C., Holmes K., Blubaugh C. (2022). Benefits and risks of intercropping for crop resilience and pest management. J. Econ. Entomol..

[92] Yang Q., Li Q., Liu X., Yang Y., Ruan Y., Zhu P., Lu Z., Cao C., Lu Y. (2026). The potential of landscape plants *Photinia fraseri* and *Pittosporum tobira* as refuge for natural enemies of pest insects in rice–wheat rotation systems. Insects.

[93] Meera K., Sreeja P., Karthikeyan K., Smitha R. (2026). Floral resources as ecological tools to enhance natural enemies for pest management in the rice ecosystem. Plant Sci. Today.

[94] Zhu P., Zheng X., Yao X., Xu H., Zhang F., Chen G., Lu Z. (2015). Ecological engineering technology for enhancement of biological control capacity of egg parasitoids against rice planthoppers in paddy fields. China Plant Prot..

[95] Zhu P., Wang G., Zheng X., Tian J., Lu Z., Heong K.L., Xu H., Chen G., Yang Y., Gurr G.M. (2015). Selective enhancement of parasitoids of rice Lepidoptera pests by sesame (*Sesamum indicum*) flowers. Bio. Control.

[96] Landis D.A., Wratten S.D., Gurr G.M. (2000). Habitat management to conserve natural enemies of arthropod pests in agriculture. Annu. Rev. Entomol..

[97] Chen G., Zhu P., Zheng X., Yao X., Zhang F., Sheng X., Xu H., Lu Z. (2016). The practice of ecological engineering on the control of rice insect pests in Jinhua. China Plant Prot..

[98] Dong Z., Yang Q., Wyckhuys K.A., Ouyang F., Gurr G.M., Han P., González-Chang M., van der Werf W., Men X., Ge F. (2026). Use of functional plants to enhance pest biological control in farming landscapes: Progress and prospects. Entomol. Gen..

[99] Tixier P., Duyck P.-F., Côte F.-X., Caron-Lormier G., Malézieux E. (2013). Food web-based simulation for agroecology. Agron. Sustain. Dev..

[100] Xu H.-X., Yang Y.-J., Lu Y.-H., Zheng X.-S., Tian J.-C., Lai F.-X., Fu Q., Lu Z.-X. (2017). Sustainable management of rice insect pests by non-chemical-insecticide technologies in China. Rice Sci..

[101] Kumar A., Nayak A.K., Sharma S., Senapati A., Mitra D., Mohanty B., Prabhukarthikeyan S.R., Sabarinathan K.G., Mani I., Garhwal R.S. (2023). Rice straw recycling: A sustainable approach for ensuring environmental quality and economic security. Pedosphere.

[102] Zhu Z., Lu Z., Yu M., Guo R., Ren D., Cheng J. (2012). Ecological engineering for pest management in rice. J. Appl. Entomol..

[103] Sun J., Song X., He J., Chen D., Yang T., Shi A. (2025). Rice stubble provides overwintering microhabitats for spiders in winter-fallowed rice fields. Agriculture.

[104] Ma X., Dai Q., Qin W., Liu J., Liu X., Chen L., Fan J., Wu M., Li D., Liu M. (2025). Milk vetch (*Astragalus sinicus* L.) affects microbial-driven rice straw decomposition in multiple stages. Plant Soil.

[105] Zheng X., Lu Y., Zhu P., Zhang F., Tian J., Xu H., Chen G., Nansen C., Lu Z. (2017). Use of banker plant system for sustainable management of the most important insect pest in rice fields in China. Sci. Rep..

[106] Stacey D. (1977). Banker plant production of *Encarsia formosa* Gahan and its use in the control of glasshouse whitefly on tomatoes. Plant Pathol..

[107] Chen X., Jaworski C.C., Dai H., Liang Y., Guo X., Wang S., Zang L.-S., Desneux N. (2022). Combining banker plants to achieve long-term pest control in multi-pest and multi-natural enemy cropping systems. J. Pest Sci..

[108] Skellern M.P., Clark S.J., Ferguson A.W., Watts N.P., Cook S.M. (2023). Banker plant bonuses? The benefits and risks of including brassicas in field margins to promote conservation biocontrol of specialist pests in oilseed rape. Insects.

[109] Busuulwa A., Revynthi A.M., Liburd O.E., Lahiri S. (2024). Banker plant efficacy to boost natural predators for management of field populations of *Scirtothrips dorsalis* Hood (Thysanoptera: Thripidae) in strawberries. Insects.

[110] Yu X., Hu C., Heong K.L. (1998). Parasitization and preference characteristics of egg parasitoids from various habitats of homopterans Kun Chong Xue Bao. Acta Entomol. Sin..

[111] Zheng X., Yu X. (1999). Dispersal patterns of natural enemies of rice planthoppers between *zizania* and rice fields. Acta Agric. Zhejiangensis.

[112] Guo X., Zhou Q.Y., Ru M.M., Cao B., Huang X., Zheng X.S., Zhu P.Y. (2024). Effects of a bank plant on the control of rice planthoppers: A field study. Ann. Agric. Crop Sci..

[113] Cortez-Madrigal H., Gutiérrez-Cárdenas O.G. (2023). Enhancing biological control: Conservation of alternative hosts of natural enemies. Egypt. J. Biol. Pest Control.

[114] Ravnjak B., Bavcon J., Čarni A. (2023). Plant functional traits of plant species colonizing forest gaps. Diversity.

[115] Miedema Brown L., Anand M. (2022). Plant functional traits as measures of ecosystem service provision. Ecosphere.

[116] Laffon L., Bischoff A., Gautier H., Gilles F., Gomez L., Lescourret F., Franck P. (2022). Conservation biological control of codling moth (*Cydia pomonella*): Effects of two aromatic plants, basil (*Ocimum basilicum*) and French marigolds (*Tagetes patula*). Insects.

[117] Amanu E., Tana T., Amsalu B., Dechassa N. (2021). Effects of maize and mung bean intercropping on performance of the component crops and system productivity. Ethiop. J. Crop Sci..

[118] Wan N.F., Zheng X.R., Fu L.W., Kiær L.P., Zhang Z., Chaplin-Kramer R., Dainese M., Tan J., Qiu S.Y., Hu Y.Q. (2020). Global synthesis of effects of plant species diversity on trophic groups and interactions. Nat. Plants.

[119] Jachowicz N., Sigsgaard L. (2025). Highly diverse flower strips promote natural enemies more in annual field crops: A review and meta-analysis. Agric. Ecosyst. Environ..

[120] Bullock J.M., McCracken M.E., Bowes M.J., Chapman R.E., Graves A.R., Hinsley S.A., Hutchins M.G., Nowakowski M., Nicholls D.J.E., Oakley S. (2021). Does agri-environmental management enhance biodiversity and multiple ecosystem services? A farm-scale experiment. Agric. Ecosyst. Environ..

[121] Kowalska J., Antkowiak M., Sienkiewicz P. (2022). Flower strips and their ecological multifunctionality in agricultural fields. Agriculture.

[122] Nichols R.N., Holland J.M., Goulson D. (2023). A novel farmland wildflower seed mix attracts a greater abundance and richness of pollinating insects than standard mixes. Insect Conserv. Divers..

[123] Jacobsen S.K., Sigsgaard L., Johansen A.B., Thorup-Kristensen K., Jensen P.M. (2022). The impact of reduced tillage and distance to field margin on predator functional diversity. J. Insect Conserv..

[124] Jacobsen S.K., Sørensen H., Sigsgaard L. (2022). Perennial flower strips in apple orchards promote natural enemies in their proximity. Crop Prot..

[125] Pfiffner L., Cahenzli F., Steinemann B., Jamar L., Bjørn M.C., Porcel M., Tasin M., Telfser J., Kelderer M., Lisek J. (2019). Design, implementation and management of perennial flower strips to promote functional agrobiodiversity in organic apple orchards: A pan-European study. Agric. Ecosyst. Environ..

[126] Rowe L., Gibson D., Landis D.A., Isaacs R. (2021). Wild bees and natural enemies prefer similar flower species and respond to similar plant traits. Basic Appl. Ecol..

[127] Mei Z., de Groot G.A., Kleijn D., Dimmers W., van Gils S., Lammertsma D., van Kats R., Scheper J. (2021). Flower availability drives effects of wildflower strips on ground-dwelling natural enemies and crop yield. Agric. Ecosyst. Environ..

[128] Tschumi M., Albrecht M., Entling M.H., Jacot K. (2015). High effectiveness of tailored flower strips in reducing pests and crop plant damage. Proc. Biol. Sci..

[129] He X., Kiær L.P., Jensen P.M., Sigsgaard L. (2021). The effect of floral resources on predator longevity and fecundity: A systematic review and meta-analysis. Bio. Control.

[130] Sun Y.X., Chen M.J., Hao Y.N., Wang S.S., Zhang C.L. (2024). Canola bee pollen is an effective artificial diet additive for improving larval development of predatory coccinellids: A lesson from *Harmonia axyridis*. Pest. Manag. Sci..

[131] Hu W., Ni K., Zhu Y., Liu S., Shao X., Yu Z., Wang L., Zhang R., Duan M., Xu W. (2025). Plant-driven effects of wildflower strips on natural enemy biodiversity and pest suppression in an agricultural landscape in Hangzhou, China. Agronomy.

[132] Mercer N.H., Bessin R.T., Obrycki J.J. (2020). Impact of buckwheat and methyl salicylate lures on natural enemy abundance for early season management of *Melanaphis sacchari* (Hemiptera: Aphididae) in sweet sorghum. Crop Prot..

[133] Horgan F.G., Crisol Martínez E., Stuart A.M., Bernal C.C., de Cima Martín E., Almazan M.L.P., Ramal A.F. (2019). Effects of vegetation strips, fertilizer levels and varietal resistance on the integrated management of arthropod biodiversity in a tropical rice ecosystem. Insects.

[134] Maas B., Brandl M., Hussain R.I., Frank T., Zulka K.P., Rabl D., Walcher R., Moser D. (2021). Functional traits driving pollinator and predator responses to newly established grassland strips in agricultural landscapes. J. Appl. Ecol..

[135] Lu Z.X., Zhu P.Y., Gurr G.M., Zheng X.S., Read D.M., Heong K.L., Yang Y.J., Xu H.X. (2014). Mechanisms for flowering plants to benefit arthropod natural enemies of insect pests: Prospects for enhanced use in agriculture. Insect Sci..

[136] Wang J.H., Ren B.B., Shao J.L., Li W., Che S.C. (2023). Impact of plant community structure and its diversity on richness and abundance of arthropod aphidophagous natural enemy communities. Urban For. Urban Green..

[137] Zhou W., Arcot Y., Medina R.F., Bernal J., Cisneros-Zevallos L., Akbulut M.E.S. (2024). Integrated pest management: An update on the sustainability approach to crop protection. ACS Omega.

[138] Rossi V., Caffi T., Salotti I., Fedele G. (2023). Sharing decision-making tools for pest management may foster implementation of Integrated Pest Management. Food Secur..

[139] Deguine J.-P., Aubertot J.-N., Flor R.J., Lescourret F., Wyckhuys K.A., Ratnadass A. (2021). Integrated pest management: Good intentions, hard realities. A review. Agron. Sustain. Dev..

[140] Scheff D.S., Phillips T.W. (2022). Integrated pest management. Storage of Cereal Grains and Their Products.

[141] Han P., Rodriguez-Saona C., Zalucki M.P., Liu S.-s., Desneux N. (2024). A theoretical framework to improve the adoption of green Integrated Pest Management tactics. Commun. Biol..

[142] Shao X., Zhang Q., Zhang B., Xie Z., Xu K. (2026). Biological control of insect pests in agroecosystems: Current challenges, innovative strategies, and future directions. Agriculture.

[143] Begum M., Gurr G.M., Wratten S.D., Hedberg P.R., Nicol H.I. (2006). Using selective food plants to maximize biological control of vineyard pests. J. Appl. Ecol..

[144] Hyder M., Ullah F., Basit A., Haq I.U., Mustapha T., Nisa Z.U., Cai X., Liu H., Hou Y. (2026). Mapping conservation biological control and IPM research (2000–2025): A bibliometric analysis of natural enemies and habitat management. Insects.

[145] Zhu Y., Chen J., Zou Y., Huang X., Jiang T., Wyckhuys K.A., van der Werf W., Xiao H. (2023). Response of the rice stem borer *Chilo suppressalis* (Walker) and its parasitoid assemblage to landscape composition. Agric. Ecosyst. Environ..

[146] Van Klink R., August T., Bas Y., Bodesheim P., Bonn A., Fossøy F., Høye T.T., Jongejans E., Menz M.H., Miraldo A. (2022). Emerging technologies revolutionise insect ecology and monitoring. Trends Ecol. Evol..

[147] Pretty J., Pervez Bharucha Z. (2015). Integrated pest management for sustainable intensification of agriculture in Asia and Africa. Insects.

[148] Deutsch C.A., Tewksbury J.J., Tigchelaar M., Battisti D.S., Merrill S.C., Huey R.B., Naylor R.L. (2018). Increase in crop losses to insect pests in a warming climate. Science.

[149] Zhu P., Lu Z., Heong K., Chen G., Zheng X., Xu H., Yang Y., Nicol H.I., Gurr G.M. (2014). Selection of nectar plants for use in ecological engineering to promote biological control of rice pests by the predatory bug, *Cyrtorhinus lividipennis*, (Heteroptera: Miridae). PLoS ONE.

[150] Babendreier D., Tang R., Horgan F.G. (2022). Prospects for integrating augmentative and conservation biological control of leaf-folders and stem borers in rice. Agronomy.

[151] Dominik C., Seppelt R., Horgan F.G., Settele J., Václavík T. (2018). Landscape composition, configuration, and trophic interactions shape arthropod communities in rice agroecosystems. J. Appl. Ecol..

[152] Baba Y.G., Kusumoto Y., Tanaka K. (2018). Effects of agricultural practices and fine-scale landscape factors on spiders and a pest insect in Japanese rice paddy ecosystems. Bio. Control.

[153] Ali M., Kabir M., Haque S., Afrin S., Ahmed N., Pittendrigh B., Qin X. (2020). Surrounding landscape influences the abundance of insect predators in rice fields. BMC Zool..

